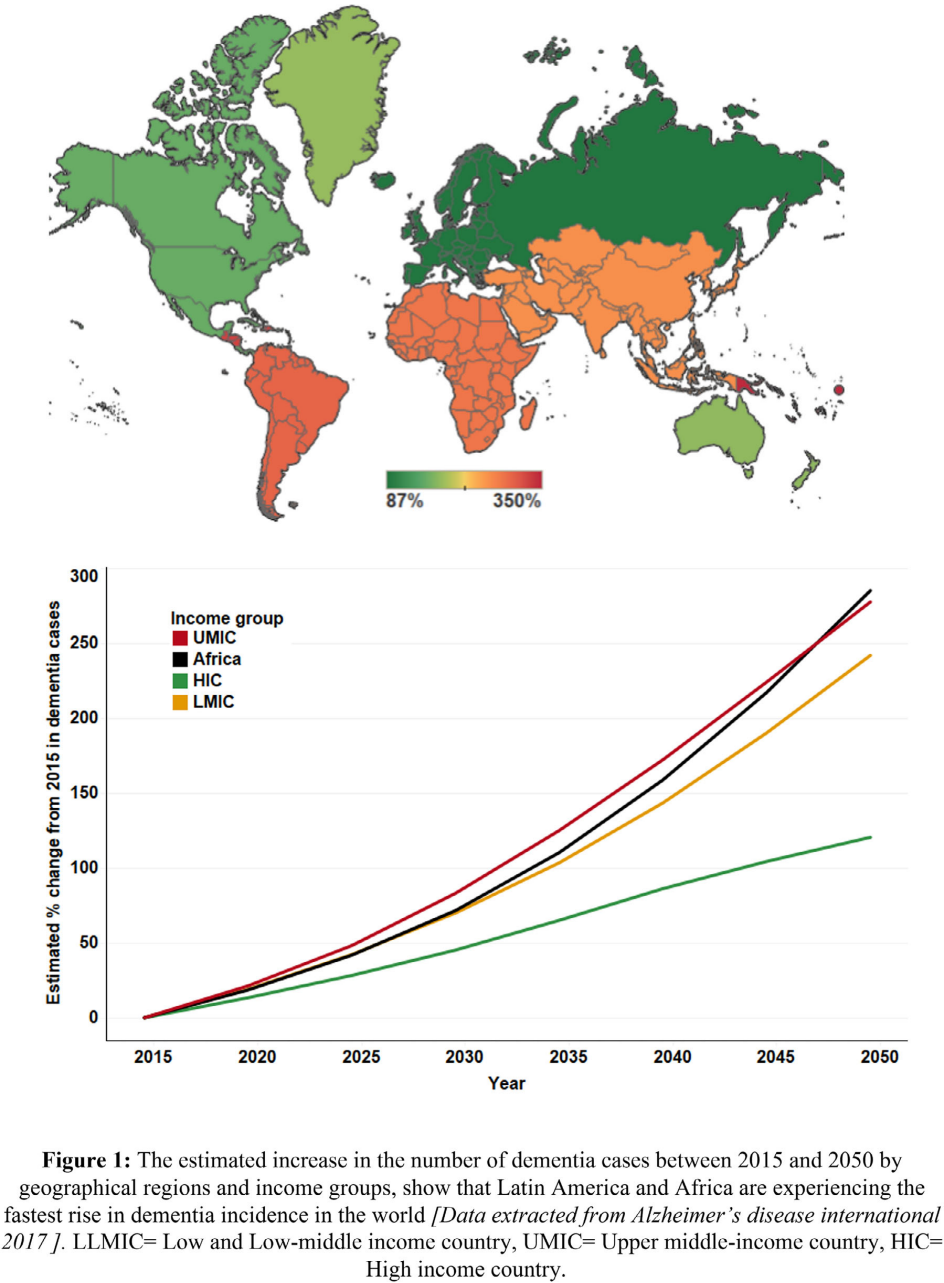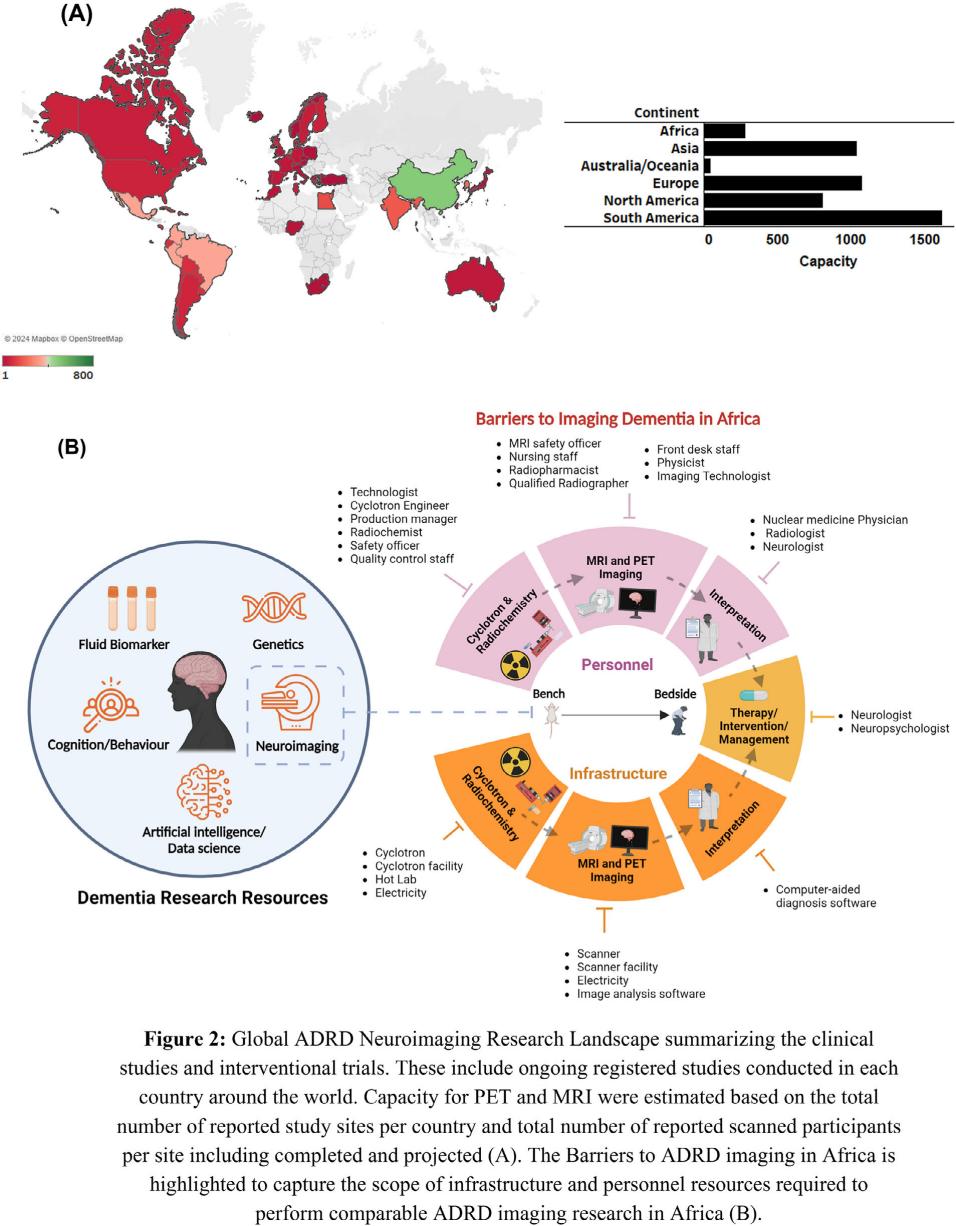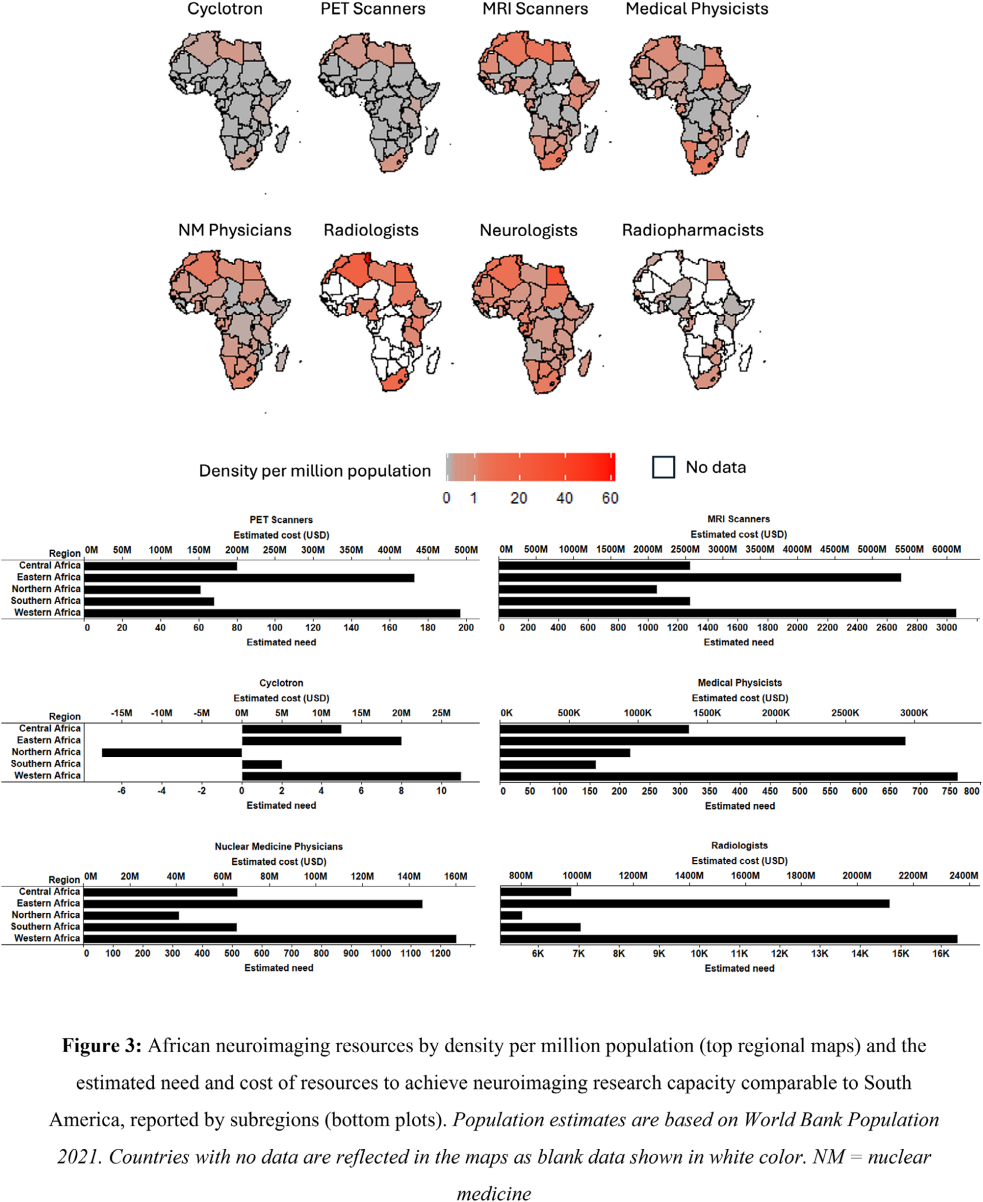# Imaging Dementia in African Populations: Closing the Health Equity Gap

**DOI:** 10.1002/alz70862_110059

**Published:** 2025-12-23

**Authors:** Olujide Oyeniran, Kirsty Donald, Akintunde Orunmuyi, Thomas K Karikari, Tavia E Evans, Jasmine Cakmak, Ethan Draper, Valentine A Ucheagwu, Justin Hicks, Pedro Rosa‐Neto, Yasser Iturria Medina, Rufus O. Akinyemi, Simon Mensah Ametamey, Linshan Liu, Sheila Waa, Chi Udeh‐Momoh, Ozioma C. Okonkwo, Udunna Anazodo

**Affiliations:** ^1^ Lawson Health Research Institute, London, ON Canada; ^2^ University of Cape Town, Cape Town South Africa; ^3^ University College London, London UK; ^4^ University of Pittsburgh, Pittsburgh, PA USA; ^5^ Erasmus MC University Medical Center, Rotterdam Netherlands; ^6^ Montreal Neurological Institute, McGill University, Montreal, QC Canada; ^7^ Montreal Neurological Institute, Montreal, QC Canada; ^8^ Nnamdi Azikiwe University Awka, Onitsha / Anambra State Nigeria; ^9^ University of Western Ontario, London, ON Canada; ^10^ McGill University Research Centre for Studies in Aging, Montreal, QC Canada; ^11^ Department of Neurology and Neurosurgery, McGill University, Montréal, QC Canada; ^12^ College of Medicine, University of Ibadan, Ibadan, Oyo State Nigeria; ^13^ ETH Zurich, Zurich Switzerland; ^14^ Lawson Research Instiute, London, ON Canada; ^15^ Aga Khan University, Nairobi Kenya; ^16^ Wake Forest University, School of Medicine, Winston‐Salem, NC USA; ^17^ Wisconsin Alzheimer's Disease Research Center, School of Medicine and Public Health, University of Wisconsin‐Madison, Madison, WI USA; ^18^ Medical Artificial Intelligence (MAI) Laboratory, Crestview Radiology Limited, Lagos Nigeria

## Abstract

**Background:**

In the next two decades, Africa is projected to have one of the highest number of people with Alzheimer’s disease and related dementias (ADRD) (Figure 1). Neuroimaging tools, particularly magnetic resonance imaging (MRI) and positron emission tomography (PET), are established diagnostic tools for characterizing ADRD. However, the use of these tools is unevenly distributed globally and acutely lacking in Africa, particularly in Sub‐Saharan Africa. Because ADRD prevalence and risk factors vary across populations and geospatial lines, there is a growing need to develop neuroimaging capacity for dementia research in diverse populations to expand our understanding of its characteristics. Here, we highlight gaps in dementia imaging research globally, identify associated barriers to their use in Africa, and provide a perspective on opportunities to enable dementia neuroimaging research across Africa.

**Method:**

We reviewed published data from literature and clinical trial databases for ongoing or past ADRD studies around the world with PET or MRI. We then estimated the number of sites, participants, and capacity (participants/site). We also used published data to estimate Africa’s imaging infrastructure and personnel needs and costs.

**Result:**

A total of 49 countries (∼25% of all countries in the world) account for the global ADRD neuroimaging research (Figure 2). On a regional level, Africa has the least number of ADRD study sites and participants who have/will have PET or MRI (Figure 2). In Africa, only 4 countries have conducted ADRD research using MRI and none have published research or reported ongoing clinical trials using PET. The challenges with acquiring and operating neuroimaging infrastructure including high costs of scanner and radiotracer production as well as shortage of skilled personnel are fundamental barriers to dementia imaging research in Africa. At minimum, $29,321,441,393 is required to equip Africa with the neuroimaging infrastructure and personnel to bridge the gap (Figure 3).

**Conclusion:**

Thus, we propose, 1) increased imaging infrastructure investment, especially in low‐cost technologies, 2) optimization of existing clinical imaging systems for advanced imaging, 3) collaborative training of local personnel through upskilling programs and 4) establishment of regional and global partnerships. Together these actions can transform dementia imaging capacity in Africa.